# Unraveling the Role of Respiratory Muscle Metaboloreceptors under Inspiratory Training in Patients with Heart Failure

**DOI:** 10.3390/ijerph18041697

**Published:** 2021-02-10

**Authors:** Hugo Fernández-Rubio, Ricardo Becerro-de-Bengoa-Vallejo, David Rodríguez-Sanz, César Calvo-Lobo, Davinia Vicente-Campos, Jose López Chicharro

**Affiliations:** 1 Faculty of Nursing, Physical therapy and Podiatry, Universidad Complutense de Madrid, 28040 Madrid, Spain; hugofern@ucm.es (H.F.-R.); ribebeva@enf.ucm.es (R.B.-d.-B.-V.); davidrodriguezsanz@ucm.es (D.R.-S.); jlopezch@ucm.es (J.L.C.); 2 Faculty of Health Sciences, Universidad Francisco de Vitoria, Pozuelo de Alarcón, 28223 Madrid, Spain; davinia.vicente@ufv.es; 3 Grupo FEBIO, Universidad Complutense de Madrid, 28040 Madrid, Spain

**Keywords:** exercise, heart failure, personalized medicine, pulmonary ventilation, respiratory muscles

## Abstract

Exercise intolerance may be considered a hallmark in patients who suffer from heart failure (HF) syndrome. Currently, there is enough scientific evidence regarding functional and structural deterioration of skeletal musculature in these patients. It is worth noting that muscle weakness appears first in the respiratory muscles and then in the musculature of the limbs, which may be considered one of the main causes of exercise intolerance. Functional deterioration and associated atrophy of these respiratory muscles are related to an increased muscle metaboreflex leading to sympathetic–adrenal system hyperactivity and increased pulmonary ventilation. This issue contributes to increased dyspnea and/or fatigue and decreased aerobic function. Consequently, respiratory muscle weakness produces exercise limitations in these patients. In the present review, the key role that respiratory muscle metaboloreceptors play in exercise intolerance is accurately addressed in patients who suffer from HF. In conclusion, currently available scientific evidence seems to affirm that excessive metaboreflex activity of respiratory musculature under HF is the main cause of exercise intolerance and sympathetic–adrenal system hyperactivity. Inspiratory muscle training seems to be a useful personalized medicine intervention to reduce respiratory muscle metaboreflex in order to increase patients’ exercise tolerance under HF condition.

## 1. Introduction

Heart failure (HF) has a high prevalence, affecting up to 23 million persons worldwide [1]. This condition generates a high impact on functional activity, quality of life and the aging process. In addition, it is a great economic burden for the healthcare system. HF is presented as a multifactorial condition that affects neuro-humoral, structural, molecular and cellular mechanisms, involving all these systems simultaneously in a network that impairs physiological function. All complex and coordinated activities may generate ventricle overload and greater sympathetic–adrenal activity, which may be associated with excessive peripheral vasoconstriction leading to blood flow limitation of locomotor system muscles. This circulation redistribution results in a complex clinical syndrome [2,3], which produces an impaired filling of ventricles with or without a reduced fraction of ejection [4], associated with a progressive deterioration of the musculoskeletal function [5,6,7]. Indeed, the pumping HF to meet the metabolic tissue demands, together with the peripheral functional impairment, may lead to dyspnea, exercise intolerance, fatigue and peripheral or lung edema [1,4,5]. HF patients suffer from a great rate of mortality and morbidity, common hospitalizations and poor quality of life [8]. This syndrome may be categorized into HF including reduced fraction of ejection (HFrEF) and HF including preserved fraction of ejection (HFpEF). Subjects with ejection fractions ≥50% are diagnosed as patients with HFpEF, while patients who present an ejection fraction from 41% to 49% are diagnosed as patients with HFrEF [1]. In addition, the New York Heart Association (NYHA) created a categorization for HF classes: class I, without symptoms or limitations; class II, slight symptoms or limitations for physical activity; class III, important symptoms or limitations for physical activity; and class IV, symptoms or limitations at resting state [9].

Exercise intolerance plays a primary role in HF patients linked to nonfavorable prognosis [10]. HF patients suffer from an altered respiratory pattern as well as dyspnea with physical activity [10,11]. In spite of the common HF sequelae, there is a lack of a clear association between exercise intolerance and cardiac functioning parameters, such as ejection fraction, volume of the left ventricle and cardiac output [10]. Hemodynamic alterations are considered key factors that produce HF symptoms secondary to heart ineffectiveness, generating an increase in cardiac output and venous pressures of systemic and pulmonary mechanisms. New evidence supports a muscular hypothesis suggesting skeletal muscle deterioration as the main cause of HF symptoms [6,11,12,13]. Muscular weakness present in HF patients occurs more commonly in inspiratory muscles than in the lower limb muscles [11,14,15]. The atrophy of these skeletal muscles is produced due to decreased cardiac output as well as tissue hypoxia processes, inflammation alterations, higher systemic catabolism and longer immobilization periods [7]. These alterations are linked to protein degradation, a higher presence of myokines and muscle fiber changes from slow-twitch or type I fibers to fast-twitch or type II fibers [5,16]. Indeed, impaired oxidative metabolism and early acidosis are produced due to a lower presence of mitochondria, decreased density of the mitochondrial ridges and reduced mitochondrial enzymatic activity decreasing enzymes such as 3-hydroxyacyl-CoA dehydrogenase, succinate dehydrogenase and citrate synthetase [16]. Consequently, these processes lead to muscular resistance reduction, afferent reflexes or meta-reflex activation, as well as sustained and increased sympathetic–adrenal activity [17,18]. Furthermore, ventilation pattern alterations produce greater dyspnea, higher fatigue and reduced aerobic capacity [5,6,7,16,17,18]. Therefore, inspiratory muscle weakness has been associated with HF leading to potential adverse effects [11,13]. Inspiratory muscle weakness is linked to greater muscle meta-reflex, playing a key role in HF prognosis [19].

Muscular meta-reflex is a blood flow regulator mechanism for cardiac output, blood pressure and regional distribution, and it involves chemical receptors of muscle parenchyma activated by metabolites secondary to muscle contractile properties [20,21]. Afferent fibers of muscle tissue involved in the meta-reflex consist of nonmyelinated group IV neurons, including chemical sensitive receptors for metabolites secondary to skeletal muscle contraction [5,20,21,22]. Currently, there is a lack of scientific evidence to determine the specific kind of metabolite that activates the muscle meta-reflex [23]. Nevertheless, potassium, lactic acid, adenosine, diprotonated phosphate, arachidonic acid, prostaglandins, capsaicin and serotonin have been suggested as possible specific metabolites that activate the muscle metaboreflex, but not for adenosine and acidic pH [20,22,24,25]. These metabolic stimuli induced by muscle contraction activate molecular receptors located at the terminal end of unmyelinated nerve fibers, such as group IV fibers which are mainly metabolosensitive. Consequently, a spontaneous discharge of the muscle afferent fibers is projected through the input dorsal root of the spinal cord and spreads to the dorsal horns of various spinal cord segments. Muscle fiber inputs reach various integration levels. These reflexes do not seem to need activity of the rostral brain, although supraspinal integration could coexist. In addition, the medulla is considered the control area for cardiovascular responses under activation of the mechano-metaboreflexes [5,26]. Efferent responses to the activation of muscular meta-reflexes may comprise higher activity of sympathetic nerves constricting systemic vessels, as well as increasing blood flow in contracted muscular fibers and provoking cardiac ionotropic responses and chronotropic effects to produce greater output at the same time. Thus, muscle meta-reflex generates sympathetic–adrenal responses, which increase blood pressure for exercise performance, allowing blood flow and volume redistribution [20].

According to the detailed process, respiratory muscle meta-reflex appears secondary to fatigue during activity that produces metabolic subproduct accumulation, triggering the activation of the receptors in unmyelinated type IV phrenic afferent fibers; projecting towards the centers of supraspinal brainstem regions implicated in the control of somatosensory cortex, hypothalamus, cerebellum and cardiorespiratory centers; and consequently provoking the meta-reflex activation of these muscles [20,21,22,27,28,29,30,31]. In addition, these meta-reflexes activate a sympathetic response, leading to vasoconstriction at a peripheral level, blood flow reduction of skeletal muscular perfusion, fatigue increase induced by exercise and redistribution of blood flow of respiratory muscles to maintain adequate functioning. Current scientific evidence suggests that one of the main reasons why the diaphragm suffers less vasoconstriction than the limb muscles is due to the fact that the diaphragm presents fewer α1-adrenergic receptors, but more evidence is required to affirm this statement [20,27,29,31,32,33,34]. The increased fatigue of skeletal muscle leads to intolerance under exercise activity and reduced strength of the muscular system [27,29]. Furthermore, respiratory muscle meta-reflex produces increased heart rates and blood pressure and reduced blood flow in renal, mesenteric and limb arterial vessels, leading to reduced O_2_ diffusion to muscles [22,25,28].

Regarding patients who suffer from HF, muscle metaboreflex presents an exaggerated activity due to key changes in skeletal muscle, leading to excessive afferent feedback to group IV receptors. One of the main causes of such exaggerated activation is the histological alteration of skeletal muscle fibers that indicates a change in the proportion of fiber type and the expression of the chain isoform of heavy myosin. These myosin chains of type I and gain of type IIx may provoke an increase in metabolic activity, a reduction of oxidative capacity and an increase in group IV afferent activation. Thus, this leads to an increase in vascular resistance at a systemic level, limiting cardiac output, stroke volume and O_2_ supply and generating impaired exercise capacity and peak VO_2_ in HF patients [35,36,37,38].

Inspiratory muscle weakness appears in approximately 50% of HF patients [30], contributing to a worse prognosis for these patients [32]. The diagnosis of HF is performed when the maximum inspiratory pressure (PI_max_) fraction is <70% with respect to the normalized value based on a patient’s age and gender [11,39] or when PI_max_ direct values are ≤60 cmH_2_O [40]. PI_max_ seems to be linked to the perception of dyspnea for daily activity, being considered as a prognostic indicator for HF patients [30,33,41,42]. Dyspnea and fatigue symptoms presented in HF patients may be partially due to respiratory muscle weakness [11,29,30,43,44,45,46] associated with decreased respiratory muscle strength, which leads to a higher PI_max_ fraction for the respiration process. Thus, patients note a higher intensity of dyspnea secondary to increased PI_max_ fractions for this process [47]. In addition, respiratory musculature weakness is linked to reduced tidal volume, which produces a higher ratio of ventilation-dead space and increased ventilation–perfusion mismatch for exercise activities performed by HF patients. Furthermore, increased correlations between CO_2_ and ventilation are presented as the main prognostic indicators for these individuals [29,45,46,47]. In conclusion, inspiratory muscle weakness increases fatigue, promoting early metaboloreceptor activation in respiratory muscles [32].

In addition, weakness of respiratory muscles may be associated with histological alterations. Respiratory muscle biopsies carried out in HF patients showed a reduced percentage in both type IIx muscle fibers and type IIa muscle fibers, as well as a greater proportion of type I muscle fibers with respect to healthy controls [7,48,49,50]. These alterations seem to differ from those presented in the skeletal musculature of limbs [7]. Although the type I muscle fiber proportions seem to commonly increase in respiratory musculature, respiratory muscle atrophy is presented in HF patients [29,39,50]. In addition, a greater proportion of type I muscle fiber is linked to higher oxidative activity of enzymes under HF conditions. All those alterations could be secondary to myogenic factors of regulation associated with an increased effort for sustained ventilation. This adaptation process may facilitate an increase in respiratory resistance, accompanied by a parallel reduction of muscular power and maximum strength for these muscles [30,48].

Considering other factors that modify respiratory functions in conjunction with inspiratory muscle weakness under HF condition, increased respiratory activity may be presented secondary to respiratory musculature because it must function against higher elastic and/or resistive loads [10]. Increased elastic load appears to be a consequence of higher stiffness of the pulmonary tissue secondary to the intrathoracic space competition between heart tissue and the lungs, such as the presence of congested pulmonary flow, cardiomegaly, bronchial vascular flows and pulmonary interstitial edema. An increase in resistive loads may lead to congestion of the lungs associated with limited expiratory flows as well as sustained hyperventilation [10,11,29], resulting in higher blood flow and oxygen requirements for respiratory muscles. HF seems to be accompanied by a limited response in cardiac output under exercise activity, and thus the fatigue appears earlier [10]. In addition, Yamada et al. [29] reported that HF patients who present respiratory muscle weakness are likely to show functional capacity limitations in 6-min walking tests, regardless of muscle weakness in the lower extremities or restrictive patterns of the respiratory system. In addition, weakness of inspiratory musculature is linked to various NYHA functional classes, with the greatest inspiratory forces found in individuals who suffer from class I HF and the lowest inspiratory forces found in class IV HF patients [30,51].

Indeed, inspiratory muscle weakness together with increased breathing load may facilitate the fatigue process of these muscles, producing early activation of metaboloreceptors of the respiratory musculature. This process causes exaggerated metaboreflex activity in the respiratory muscles of HF patients. It should be noted that this increased activity may also produce higher hyperventilation in these patients, since the afferents of group IV neurons through dorsal horns of spinal cord neural structures reach respiratory centers of the medulla, such as the rostral ventral cord, caudal ventro-lateral cord and solitary tractus nucleus, generating an exacerbated fatigue of the respiratory muscles [10,32]. Furthermore, muscle metaboreflex, in conjunction with weakness of these muscles, may be considered an important activator of chemoreflex, through peripheral and/or central stimuli, under HF condition. This provokes activation under a lower threshold, which leads to sustained increases in sympathetic activity and causes adrenergic vasoconstrictions, as well as an increase in right and/or left ventricular afterload. Thus, sympathetic hyperactivation is considered an important mortality predictor in HF patients [32].

An interesting method used to investigate respiratory muscle fatigue and its consequent activation of the metaboreflex in HF patients with exercise intolerance is the use of a noninvasive mechanical ventilator to reduce the inspiratory muscle load [52,53,54,55]. In these studies, the load reduction of these muscles increased blood flow of limbs, cardiac output, muscle oxygen absorption (VO_2_), muscle oxygen diffusing capacity (DMO_2_) and exercise tolerance and decreased the vascular resistance of limbs and sympathetic–adrenal system activity. Respiratory muscle metaboreflex needs the limbs’ blood flow since the discharge of the inspiratory muscles produces increased blood flow in the extremities and a reduction of blood flow in the respiratory musculature [52,53,54,55]. Therefore, it seems that inspiratory musculature training (IMT) could be presented as a useful therapy in order to decrease the respiratory muscle metaboreflex, which could reduce fatigue of these muscles [56].

## 2. Material and Methods

### 2.1. Study Design

A study design of narrative review was used according to “Preferred Reporting Items of Systematic Review & Meta-analyses (PRISMA)” guidelines [57]. The present study was carried out in order to provide an update of available information of previous systematic reviews considering the reduction of respiratory muscle metaboreflex by IMT in HF patients [42,58], including various study types such as quasi-experimental studies and clinical trials with publication dates before September 2020. 

### 2.2. Search Strategies

A database search was performed in September 2020. PubMed was the database used for this search process. Used restrictions were experimental records providing full access by the search strategy named as (“inspiratory muscle training” OR “respiratory muscle training”) AND “heart failure” included in abstract and/or title of English and/or Spanish language studies [56].

### 2.3. Study Selection

Inclusion selection criteria consisted of experimental records of different types, such as clinical trials and/or quasi-experimental studies analyzing the effects of IMT on respiratory muscle metaboreflex in HF patients. Exclusion criteria included nonexperimental research studies and meta-analyses or systematic reviews that comprised patients with comorbidities in conjunction with IMT such as stroke, lung hypertension, Fontan circulation and mitral valve pathologies, among other conditions, as well as all studies that did not analyze IMT effects on respiratory muscle metaboreflex. All possible outcome measurements analyzing IMT effects in an isolated way or in conjunction with other treatments on metaboreflex of respiratory muscles were included in this review in order to update the available information on HF patients [42,58].

### 2.4. Data Extraction Processes

Research features including sample sizes, demographic and social data, baseline outcome measures, training protocols, detailed schedule, frequencies, intensities, IMT devices and protocols were recorded and are detailed in Tables 1 and 2.

Data extraction included study citations, intervention groups, sample size, mean ± standard deviation (SD), *p*-value to detail statistical significance and supplemental information on measurement procedures; these results are detailed in Tables 1 and 2 following the updated review studies [42,58].

## 3. Results and Discussion

### 3.1. Flow Chart

**Figure 1:**
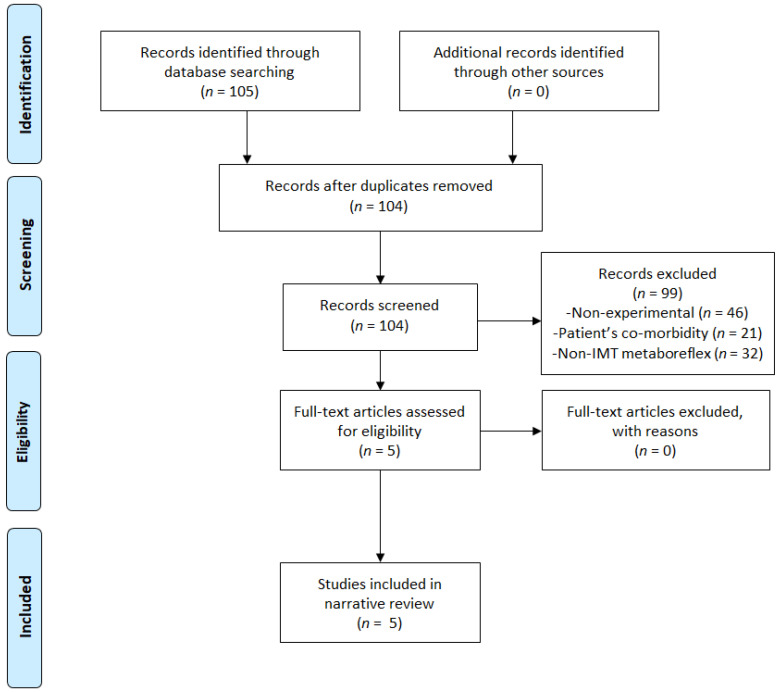
Flow chart.

From 105 studies that were identified by search processes, 100 records were removed according to exclusions and duplicates. Lastly, five records were selected for the narrative synthesis, as shown in Figure 1.

### 3.2. IMT Effects on Metaboreflex in HF Patients

IMT is a training intervention using workloads for inspiration [42]. IMT may be carried out utilizing three different devices such as inspiratory threshold, resistive load and isocapnic hyperpnea devices. Firstly, IMT by inspiratory threshold device uses inspiratory pressures to provoke the openness of a valve allowing air flows to pass for the inspiratory process. Secondly, IMT by resistive load device uses several holes of different reduced diameters which generate resistance for the inspiratory process. Thirdly, IMT by isocapnic hyperpnea device uses an equipped laboratory of respiratory physiology to maintain a ventilation level for volitional hyperpnea for 12 min while the CO_2_ is combined with the inspiration air maintaining isocapnia status for the arterial blood flows [59].

Inspiratory muscle weakness presented in HF patients may be reversible. Therefore, IMT is a useful intervention for improving the strength and/or resistance of inspiratory musculature in HF patients [56,60].

Currently, a total of 30 papers have studied the IMT effects in HF patients [28,39,40,44,61,62,63,64,65,66,67,68,69,70,71,72,73,74,75,76,77,78,79,80,81,82,83,84,85,86]. From these 30 studies, only 5 studies [28,66,67,74,85] analyzed the IMT effects on respiratory muscle metaboreflex. Four studies were randomized clinical trials [28,66,74,85] and one study was a quasi-experimental study [67] (Table 1). Sample sizes were small for most of these studies. All studies were performed in HFrEF patients [28,66,67,74,85]. IMT modality applied in two studies was inspiratory training with threshold devices [67,74], and two studies applied resistive load devices [66,85]. Concretely, Moreno et al.’s [28] study provided the IMT intervention group with the possibility to apply devices for resistive load or threshold (Table 2). 

Cardiac rehabilitation may be considered a comprehensive rehabilitation program that improves functional capacities under HF condition. Currently, cardiac programs for rehabilitation seem to be underutilized [87]. IMT intervention is a useful alternative therapy that is suitable for the participation of HF patients. In addition, IMT may be adequate for patients who are not candidates for cardiac programs for rehabilitation, such as individuals unable to carry out exercises, so it is considered a useful personalized medicine intervention [42,58]. Inspiratory muscle weakness presented in HF patients may be reversible. 

Regarding metaboreflex activity, five studies [28,66,67,74,85] detailed IMT effects on respiratory muscle meta-reflexes; statistically significant improvements (*p* < 0.05) were claimed in four of them, but such improvements were not found in the study performed by Laoutaris et al. [66]. A total of three studies [67,74,85] analyzed IMT effects on blood flow of limb muscles for rest and exercise activity. These studies reported improvements in this parameter due to vascular resistance reduction in limbs, except for Laoutaris et al.’s [66] study, which did not observe any statistically significant difference (*p* > 0.05) in the blood flow of forearms between intervention groups, although the reactive hyperemic blood flow of forearms showed statistically significant improvement (*p* < 0.05) due to IMT intervention. IMT produces statistically significant increases (*p* < 0.05) in ventilation loads required in order to produce peripheral vasoconstrictions by the respiratory musculature meta-reflexes. Such a fact could be explained by higher fatigue resistances in those muscles, producing a reduction in metabolite accumulation that triggers activation of the meta-reflexes [28,67,74,85]. Therefore, IMT may be linked to an inspiratory muscle release effect by greater inspiratory muscle thickness, diaphragmatic muscular aerobic capacity and strength. These effects were linked to a reduction of the accumulation of metabolites as well as decreased peripheral vasoconstriction, leading to greater peripheral blood flows as well as higher exercise tolerance [27,46,67]. Following the reported meta-reflexes reduction, Mello et al. [74] detailed that IMT decreased muscular sympathetic activities, producing an improvement of the vagal and sympathetic modulations for cardiovascular systems under HF condition. In addition, the reduction of the sympathetic adrenal activity may be explained as secondary to the chemo-reflex reduction due to IMT provoking an improvement in oxygen saturation. Indeed, Moreno et al. [28] detailed how respiratory muscle meta-reflexes were reduced by IMT intervention. In conclusion, an improvement in the oxygen saturations for forearm and/or intercostal musculature was reported, improving the muscles’ O_2_ diffusion. Thus, IMT may be a suitable intervention to decrease the inspiratory musculature meta-reflexes under HF condition, which would be a key intervention option for personalized medicine in HF patients.

### 3.3. Future Research and Limitations

Although statistical significance and data extraction were accurately detailed, methodological quality analyses, risks of bias, heterogeneity analysis and forest plots were not included because confidence intervals were not detailed for all studies. Our study’s records showed heterogeneity and did not permit us to perform systematic reviews and/or meta-analyses. These issues justified this narrative review to detail the available scientific evidence about IMT effects on respiratory muscle metaboreflex activity of HF patients. Furthermore, the present narrative review included the available experimental records, including clinical trials as quasi-experimental records that analyzed IMT effects on respiratory muscle metaboreflex in HF patients. Future research studies should perform high-quality meta-analyses and systematic reviews following the PRISMA criteria [57] to provide updated strong evidence about IMT effects on respiratory muscle metaboreflex in HF patients [42,58].

It is worth noting that the accuracy of distinguishing between primary or muscular and vascular induced HF or weakness may present some difficulties because the proposed methods seem to be less suitable for this differential diagnosis, as discussed by Guski et al. [88]. These authors proposed the use of specific antibodies to provide information about the immunoreactivity of ischemia-damaged cardiomyocytes. In addition, some future studies should consider virtual autopsy for HF prediction because this method may offer additional benefits for digital tissue-based diagnosis, such as tissue or organ dysfunctions and the HF patient’s prognosis [89].

## 4. Conclusions

Currently available scientific evidence seems to affirm that excessive metaboreflex activity of respiratory musculature under HF condition is one of the main causes of exercise intolerance and sympathetic–adrenal system hyperactivity. IMT seems to be a useful personalized medicine intervention to reduce respiratory muscle metaboreflex in order to improve exercise tolerance in HF patients.

## Figures and Tables

**Table 1:** Characteristics of the studies.

Year and Author	Group (*n*), Male/Female	Age (Years)	NYHA Class (II/III)	LVEF (%)	PI_max_ (cmH_2_O)
2008. Chiappa et al. [67] *	IMT: 18, 12/6 C: 10, 8/2 (Healthy subjects)	IMT: 57 ± 11 C: 38 ± 12	IMT: 10/8 C: N/A	IMT: 24 ± 3 C: N/A	IMT: 60 ± 8 C: 153 ± 26
2008. Laoutaris et al. [66]	IMT: 14, 11/3 C: 9, 9/0	IMT: 53.4 ± 2.1 C: 57.3 ± 4	IMT: 9/5 C: 6/3	IMT: 28.9 ± 2.4 C: 28.6 ± 1.9	IMT: 78.5 ± 4.9 C: 84.6 ± 5.9
2012. Mello et al. [74]	IMT: 15, 9/6 C: 12, 5/7	IMT: 54.3 ± 2 C: 53.3 ± 2	IMT: 15/0 C: 12/0	IMT: 33.6 ± 2.3 C: 37.6 ± 1.6	IMT: 56.1 ± 2.3 C: 56.2 ± 2.1
2017. Moreno et al. [28]	IMT: 13, 8/5 C:13, 8/5	IMT: 61 ± 14 C: 60 ± 13	IMT: 6/7 C: 7/6	IMT: 35 ± 9 C: 37 ± 6	IMT: 60 ± 13 C: 60 ± 16
2020. Antunes Correa et al. [85]	IMT: 11, 3/8 C: 10, 6/4 AET: 12, 7/5	IMT: 55 ± 3 C: 57 ± 3 AET: 57 ± 2	IMT: 8/3 C: 9/1 AET: 9/3	IMT: 31 ± 2 C: 25 ± 1 AET: 26 ± 2	IMT: 86 ± 9 C: 85 ± 8 AET: 87 ± 10

AET = aerobic exercise training; C = control group; IMT = inspiratory muscle training; LVEF = ejection fraction left ventricle; N/A = no available data; NYHA = New York Heart Association; PI_max_ = maximum inspiratory pressure. * Quasi-experimental study.

**Table 2:** Description of the interventions.

Year and Author	Type of IMT	Intensity of IMT	Duration per Session (min)	Frequency per Week (days)	Duration of Intervention (weeks)	Extra Commentary
2008. Chiappa et al. [67] *	Threshold device	IMT: 30% PI_max_	IMT: 30	IMT: 7	4	Control group without intervention. In the experimental group, the intensity was readjusted weekly and one session weekly was supervised.
2008. Laoutaris et al. [66]	Resistive load device	IMT: 60% SMIP C: 15% SMIP	IMT: N/A C: N/A	IMT: 3 C: 3	10	All sessions were supervised. In the experimental group, the intensity was readjusted in each session; in the control group, it was fixed. The session had six efforts at each level: Level I: 60 s for rest in each inspiration effort Level II: 45 s for rest between series Level III: 30 s for rest between series Level IV: 15 s for rest between series Level V: 10 s for rest between series Level VI: 5 s for rest between series. After level VI, rest for 5 s maintained up to respiratory fatigue process.
2012. Mello et al. [74]	Threshold device	IMT: 30% PI_max_	IMT: 10min x 3/day	IMT: 7	13	Control group had usual care. In the experimental group, the intensity was readjusted weekly and one session weekly was supervised.
2017. Moreno et al. [28]	Threshold or resistive load devices	IMT: 30% PI_max_	IMT: 30 min	IMT: 6	8	Control group without intervention. In the experimental group, the intensity was readjusted weekly and one session weekly was supervised.
2020. Antunes Correa et al. [85]	Resistive load device	IMT: 60% PI_max_	IMT: 30 min AET: 60	IMT: 5 AET: 3	16	Control group without intervention. In the experimental group, the intensity was readjusted weekly and one session weekly was supervised. In the AET group, all sessions were supervised. Sessions comprised 5 min for stretching exercise, 40 min for cycling, 10 min for local strengthening exercise and 5 min for cooldown. Aerobic exercise was performed under anaerobic thresholds up to 10% under respiratory compensation points.

AET = aerobic exercise training; C = control group; IMT = inspiratory musculature training; N/A = no available data; SMIP = sustained maximum inspiratory pressure. * Quasi-experimental study.

## Data Availability

Data will be available upon corresponding author request.
